# Tracing affordances: mixed-methods review on techniques to study affordances in virtual reality environments

**DOI:** 10.1007/s10339-025-01280-x

**Published:** 2025-06-06

**Authors:** Tania Miranti Chumaira, Lily Díaz-Kommonen, Luis Emilio Bruni

**Affiliations:** 1 Aalto University, Otaniementie 14, 02510 Espoo, Uusima Finland; 2 Aalborg University, A.C. Meyers Vænge 15, 4-015, 2450 Copenhagen, Denmark

**Keywords:** Virtual reality, Mixed-methods review, Affordance-based measurements, Architectural methods, Reading spaces, Reading architecture

## Abstract

The development of virtual reality (VR) research and innovation has mainly revolved around graphic enhancement and novel ways of human-computer interaction. In recent years, many VR researchers have urgently started to investigate methods to assess *elements* of the spatial experience of VR, such as presence and affordances. In the recent two decades, while VR researchers began to learn to measure such elements within the VR environment, studies of these elements have already been common in architecture, although the methods might differ. Therefore, this study reviews different techniques to study affordances in architecture and VR research through a mixed-method review. First, we conducted a systematic review on the methods used to study affordances in VR environments. Then, we proceeded to undertake a traditional literature review on those methods which assess spatial affordances in architecture. This study identifies the merits of current techniques of measuring affordances in both physical and virtual spaces. Through this study, we would like to suggest three methods employed in architecture as an alternative with which to assess affordances when studying spatial experience in VR environments.

## Introduction

Since Gibson’s^1^ seminal theory of affordances (1979), the concept of affordances has been developed and applied in a variety of fields that go for example from experimental psychology to human-computer interaction, industrial design, and architecture (Burte 2008; Norman 1988). In the past two decades, there has been a growing interest in utilising the concept of affordances to evaluate Virtual Reality spaces in order to observe whether the user gets the experience intended by the designers. Prior to this relatively new theory adoption, studies in VR research evaluation primarily had been conducted by implementing “sense of presence” measurements. With the advanced development of the theory, many scholars argue (Brown et al. 2015; Dourish 2006; Memikoğlu and Demirkan 2020; Stoffregen et al. 2015) that implementing the concept of affordances in studying VR environments could help designers to unfold the reciprocity between the VR users, the VR environments, and the virtual objects.

On the other hand, the practice of studying and measuring space has been done in architecture for decades, commonly known as “reading space”. Architects study, assess, and measure space through first-person methods that position the body as the measuring instrument, such as model-making, movement notation, and creative mapping. These three methods enable architects to freely explore spaces and characterise the subject’s spatial experience with different types of representations, such as three-dimensional models, diagrams, and maps (Halprin 1970). Therefore, there is a potential to gain valuable insights into how humans perceive affordances in space through researching the techniques to read space in architecture.

In this article we are going to relate two different domains that may have much more in common than what has been up to now acknowledged, namely VR research and architecture. In architectural research, the concept of reading space has been very influential in developing methodologies for the *studying of the affordances* implicit in our relation to the space (Brooker and Stone 2007). Since there has been a growing interest in the theory of affordances in fields related to Virtual Reality (VR) design, we suggest that there is a need to review and relate the literature on affordances and ways to assess or measure affordances both in VR research and architecture.

In this direction, the main contribution of the present article is to bring together these two different domains, which have not previously been systematically related, in order to draw analogies that can allow the transfer of knowledge, insights, and methods from one domain to the other. Towards this aim, we conduct a mixed methods review in which a systematic review and a traditional literature review are conducted, respectively. We adopt a systematic review to explore the literature on techniques to measure affordances within the VR research domain. Then, we resort to a traditional literature review to summarise the topic in the architecture domain. This systematic research identifies the existing techniques and approaches to measure and analyse affordances in VR research, in which the focus remains on the quantitative approach and replication of Warren and Whang’s (1987) aperture simulation. Furthermore, such a quantitative approach sees the human experience as points of events, dismissing the linkage and continuity between each point event. Meanwhile, the traditional literature review illuminates the potential of architectural techniques in gathering insights into the narrative of subjective human experience, allowing a better understanding of the causes of their experience and the way each point of event influences each other. Such findings point to actual research gaps and support our assumptions about potential insights coming from the affordances measurements techniques in architecture. In this way, the findings have also demarcated the potential future work of the study, which is to explore the affordances measurements in virtual reality environments (VREs) in an interdisciplinary manner.

We hope that our findings enrich the discussion on potential techniques to measure and analyse affordances in VREs by discussing the research gaps with available techniques from a domain outside VR research. By better understanding how to measure and analyse affordances in VREs, designers could have better knowledge of factors that shape affordances and ways to incorporate these factors into design. Thus, such a piece of knowledge could optimise the creative design processes, leading to more usable and engaging design (Dourish 2006). To the best of our knowledge, this study is the first attempt to review techniques to measure affordances in VREs by relating VR research to architectural domains.

The article is organised according to the order of the study, consisting of six sections: abstract, introduction to the topic, methods, systematic review in VR research domain, traditional literature review in architecture domain, discussion on insights and findings, and conclusion.

## Methodology

This study reviews the current state of research on affordance-based measurement techniques as tools to observe the users’ experience in VR environments by conducting a mixed-methods review that explores existing studies in VR research and architecture. This mixed-methods review consists of two separate reviews: an interdisciplinary systematic review on VR research and a traditional literature review of the literature on architecture. Two different review methods were conducted to optimise literature search processes. Each review component will be discussed in more detail below.

The first component of this mixed-methods review is an interdisciplinary systematic review in the field of VR research since VR applications have revolved around numerous fields of knowledge, such as neuroscience, psychology, computer science, industrial design, and architecture. A systematic review was selected to study the techniques for conducting affordance-based measurements in VR environments due to the need to collate all known knowledge on the topic area, identify emerging discussions, and observe current state-of-the-art research (Grant and Booth 2009). As this research involves the development of specific and relatively new technology, it is essential to acknowledge the progress of the discourse and advancement of the research within the topic (Kitchenham 2004; Yung and Khoo-Lattimore 2019).

To conduct this systematic review in the field of VR research, a literature search^2^ was carried out using the Google scholar search engine by employing strings, such as “perceiving affordances”^3^ AND “virtual reality” AND body in architectural space^4^ (see Table 1 for a detailed process). Such keywords narrow the literature search process, which discusses the experiences of the affordances in VR environments. In addition, we also wrote in *body in architectural space* to expect returns discussing the topic of the way human body relates to space as well as literature or theories derived from architecture. From the search, we received 104 returns. We then filtered the work by critically reading the abstract to evaluate the relevance of the studies to our research aim. In this study, we focus on discussing works that position VR environments as an alternate place or universe where active interaction between humans and space can occur differently compared to our actual environment. Thus, we omit works that use virtual reality as modes of representation, in which studies have already presumed that many aspects of the spatial dynamics would be similar to our actual environment. Of the 104 studies, we sorted 22 studies (details of the studies will be summarised in Table 4 in Sect. 3) that seemed most pertinent to our research aim and which were included in our systematic review. Finally, we synthesised and coded the sorted works into a spreadsheet. All steps within the systematic review follow the systematic quantitative research process model presented by Yang et al. (2017)^5^ as illustrated in Table 2 on the following page.

**Table 1:**
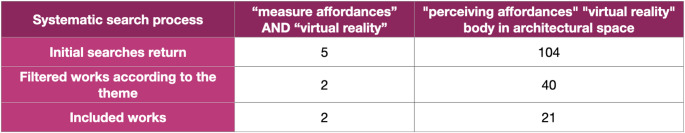
The general statistics of the systematic search

**Table 2:**
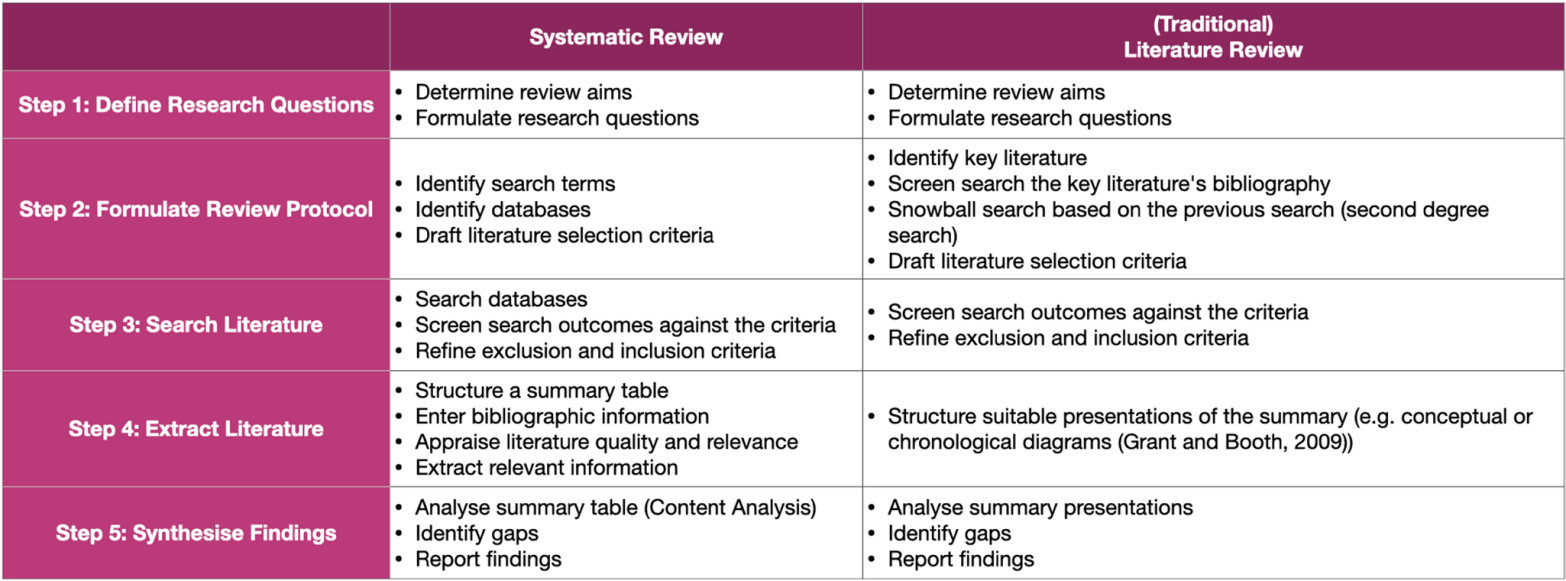
Steps in conducting the mixed-methods review

The second component of this mixed-methods review is a traditional literature review^6^ in the field of architecture. The process of this review began with a review of the initial seed studies that provide the answers to our preliminary research questions, such as: (i) How is the concept of affordances understood and used in architecture, (ii) How is the concept of affordances assessed and analysed in architecture, and (iii) What are the methods to study affordances in architecture. Departing from such questions, we identified five initial critical studies, as illustrated in Table 3.

The identified studies provide some insights into the research topic, such as: (i) in architecture, the practice of assessing or studying affordances is similar to reading spaces, which is understood as an act of understanding the relationships between the qualities of spaces and the way people adapt to spaces(Brooker and Stone 2007; Harahap et al. 2018), (ii) methods for communicating design in architecture are often used as methods for reading spaces (Brooker and Stone 2007; Halprin 1970), (iii) the most common methods for communicating design in architectural design are sketching, model-making, movement-notation, and mapping (Halprin 1970), as well as (iv) the earliest and the most recent studies on affordance-based measurement or analysis in architecture and urban studies (Burte 2008; Jelić et al. 2016). These five studies enabled a further snowball search to help us recognise the general discussion of the topic. In addition, the identified methods from the pivotal studies provided more ideas to conduct a more specific literature search on sketching, model-making, movement-notation, and mapping. After the search process, we compiled the relevant information into a spreadsheet to help us synthesise our findings. All the processes can be found in Table 2 above.

**Table 3:**
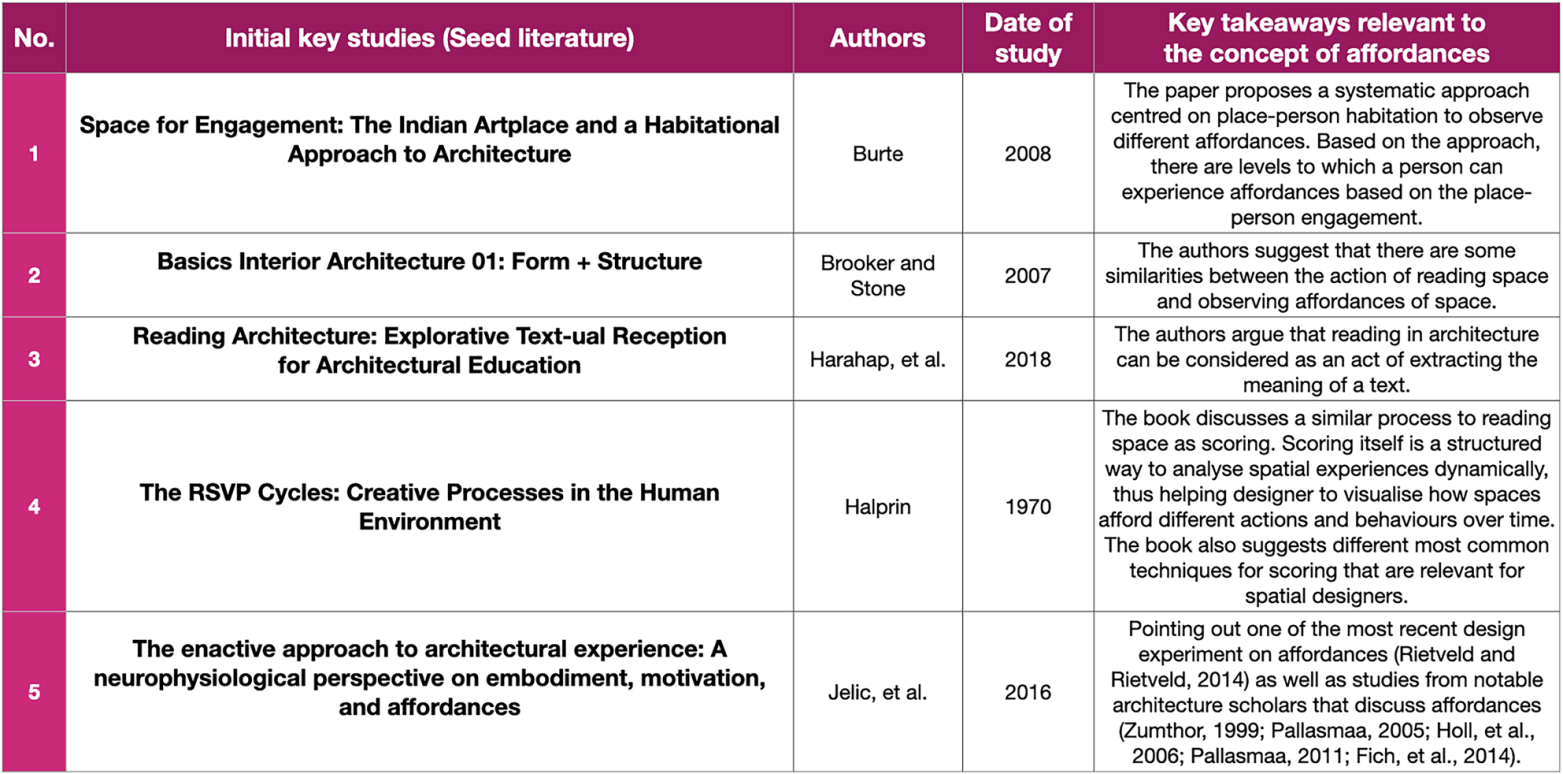
List of the seed papers that inspire the discussion about affordances and how they are typically assessed in architecture

Both approaches have their own merits and limitations. For instance, a literature search through a search engine might overlook some similar and significant literature that does not have the exact keywords. At the same time, it is more challenging to limit the scope of a conventional literature review. In addition, a conventional literature review cannot provide a legitimate justification of the state-of-the-art research in the same way as a systematic review.

This study is the first interdisciplinary review under the theme of techniques for measuring affordances that involve VR research and architecture. Thus, we also acknowledge its possible limitations, which may have overlooked some existing works. Nevertheless, affordances in space are one of the topics that have long been discussed in architecture. Therefore, we envision that this work might significantly contribute to research on techniques for measuring affordances by critically discussing the merits and drawbacks of the current techniques for measuring affordance in architecture and VR research.

## Systematic review in VR research domain

This section discusses the insights gained from our systematic review on methods that utilise affordance-based measurements in the VR research domain. We organise the discussion according to the emerging themes identified during the systematic search and filtering process, ranging from the prevalence of quantitative approaches in analysing affordances in VREs. Additionally, we reflect on the identified themes as it points to a suggestion to look into the methods used in architecture to analyse affordances in spaces.

### General trends in the discussion of affordances’ measurement

As mentioned in Sect. 2, the systematic review discusses the emerging themes and debates that have been identified from the selected 22 studies (the list of the studies can be found below in Table 4). We classified such studies based on three general themes: (1) the type of the studies, (2) the domain of the first authors^7^, and (3) the general approach of the studies. We will explore more about these classifications in the following paragraphs.

The first classification is driven by the domain of the studies. Table 4 below illustrates the distribution of the studies according to the research domains. The table is equipped with other relevant information on the studies, such as the type of the studies, the selected approaches, as well as the dates of the studies. Furthermore, the table is also organised according to the dates of the studies, showing fields that have contributed to the topic discussion for almost two decades as well as some new fields that are relatively new to the discussion.

**Table 4:**
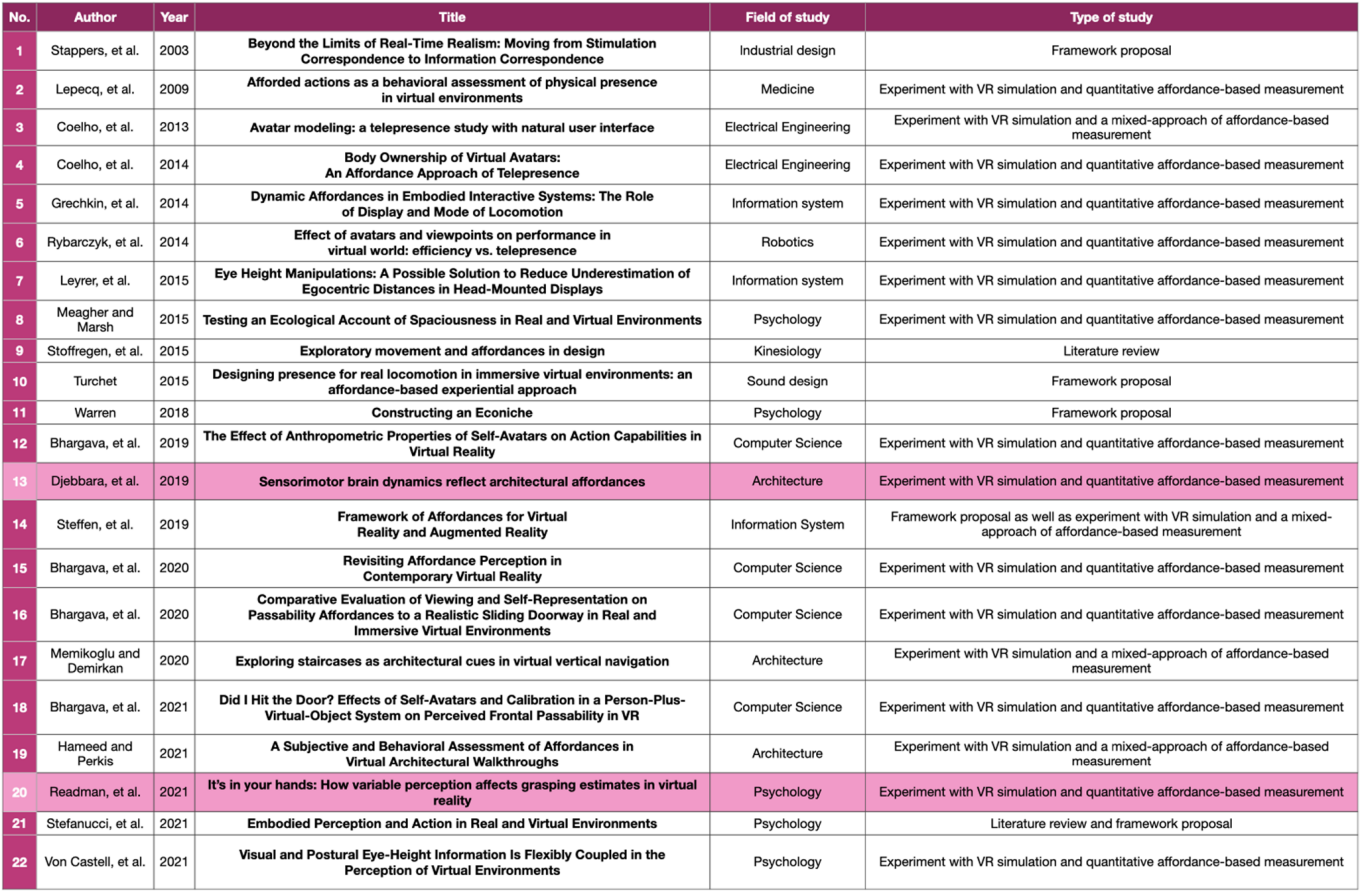
List of the included studies for the systematic review

**Fig. 1:**
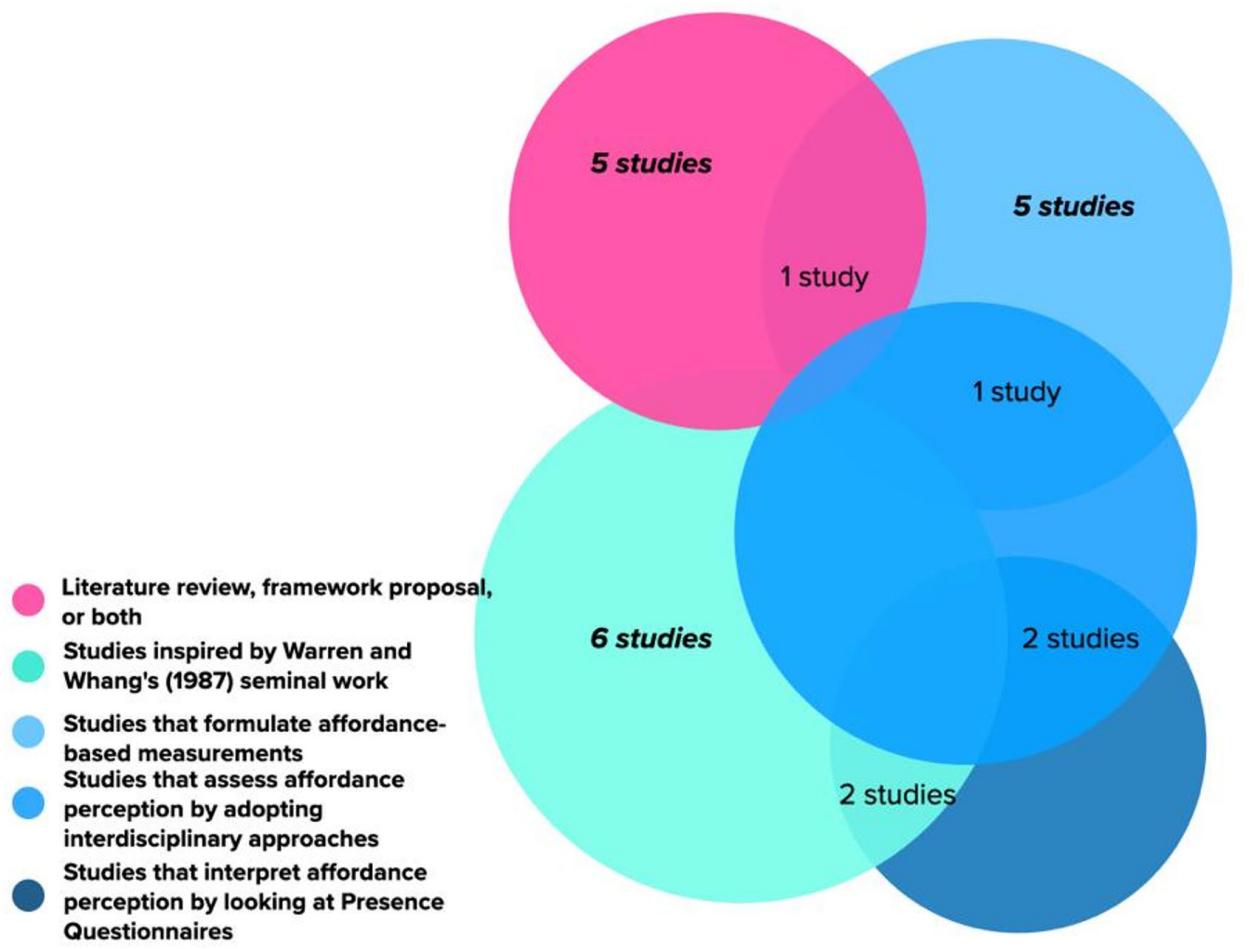
The classifications of the included studies based on their types and approaches

The second classification of the studies is organised according to the types of studies as illustrated by Fig. 1 on the next page. Figure 1 illustrates two main classifications of the studies: (1) literature review, framework proposal, or both, and (2) studies that involve VR simulation and affordance-based measurements. The first cluster is represented by the pink shade, while the blue shade represents the second cluster. By taking a closer look at the second group, we have identified several trends from the studies, such as (1) eight studies implement Warren and Whang’s (1987) affordance-based measurements that are indicated by human shoulder rotation when passing apertures in order to assess the immersion or the sense of presence, (2) seven studies attempt to formulate their affordances-based measurement metrics to specifically study the users’ perceived affordances, (3) three studies interpret correlates of affordance perception by adopting interdisciplinary theories, such as transitions in architecture (Djebbara et al. 2019), wayfinding and navigation (Memikoglu and Demirkan 2020) and the pragmatic and hedonic model of experience design (Hameed and Perkis 2021), (4) Four studies interpret correlates of affordance perception from existing Presence Questionnaires, and (5) Six studies belong to more than one classification. Even though many studies still orientate to the affordance-based measurement coined by Warren Jr and Whang (1987), there has been a growing interest both in utilising the concept of affordances as a way to assess the immersion and the sense of presence as well as formulating an affordance-based measurements as a way to study user experience in VR spaces.

Subsequently, the third classification was organised to observe the primary approach of the experimental studies. From Fig. 1, we can notice that if there are five studies discussing literature review, framework proposal or both, it means that there are remaining 17 experimental studies that attempt to assess affordances in VREs. Of the 17 studies (see Table 5 below), we have identified that the majority of the studies (76,5%) employ a quantitative approach to study correlates of perceived affordances.

**Table 5:**
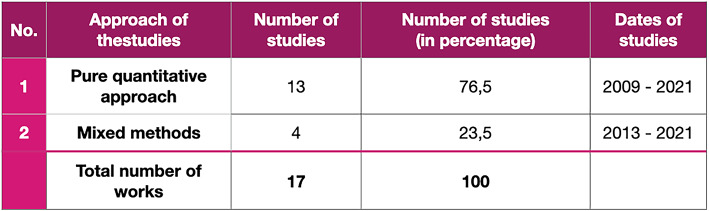
Statistics of the affordance-based measurements categorised by the approaches

The general statistics of the studies above reflect several emerging themes that could help us to better understand what has and has not been discussed within the discussion of affordances’ measurements in VREs. These emerging themes consist of (1) the approach to study affordances through presence questionnaires and (2) the recent novel approaches to measuring affordances, such as adopting interdisciplinary theories and implementing mixed-methods. Such themes will be thoroughly discussed in the two subsequent subsections.

### Studying affordances through presence questionnaires

Another trend that has been identified from the systematic review is that several studies correlate the sense of presence (as measured by presence questionnaires) to different kinds of affordances or variables related to affordances. Four out of 17 experimental studies followed the standard procedure of post-testing by investigating people’s experiences of VREs through VR simulation and then assessing the quality of their experience through questionnaires. These four studies involved the sense of presence measurement as a part of their research (see Table 6). The metric used for the presence measurement varies between the Witmer and Singer (1998) questionnaire the ITC SOPI (Lessiter et al. 2001) and the Igroup Presence Questionnaire (Regenbrecht and Schubert 2021).

**Table 6:**
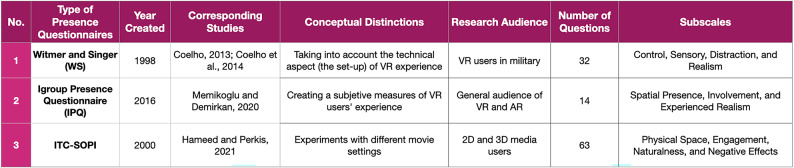
Different presence questionnaires used to study affordances in VR spaces

In general, presence is defined as the sense of being in a certain place (Slater 2009). The sense of presence is an important concept in VR experiences, as it suggests an immersive experience that distinguishes the VR medium from mediums such as cinema, where a person watches a movie and is dissociated from the visual experience. Although the identified presence questionnaires above attempt to measure the same thing, which is the sense of presence, the questionnaires are formulated for different purposes and mediums. For example, Witmer and Singer (1998) elaborated further on the subfactors as such: (1) control factors consist of the degree of control, immediacy of control, anticipation of the event, mode of control, and physical environment modifiability, while (2) distraction factors consist of isolation, selective attention, and interface awareness. While these questionnaires take into account complex aspects of the VR user experience, they are criticised for including subfactors that depend on the dexterity of VR users in using the VR system (Slater 1999), arguing that this makes the questionnaire limited only to VR contexts (Lessiter et al. 2001).

In the context of understanding affordances in VREs, such Presence Questionnaires position affordances as essential elements that construct the sense of presence. Therefore, those studies hypothesise that the more affordances (action possibilities) found within the VR experience, the more profound the sense of presence will be. Thus, the *concept of affordances* can be incorporated as factors that can be easily adjusted.

Such thoughts on the relationship between affordances and a sense of presence align with Grabarczyk and Prokropski’s (2016) conclusion. They have conducted a literature review that connects the dots between presence, affordance perception, and embodiment. Through observing the similarity between seminal works conceptualising presence, Grabarczyk and Prokropski (2016) identified two critical findings related to affordances. The first is that affordances and embodiment are essential determinants of a sense of presence. The second one is that affordances can be measured and quantified. Therefore, analysing affordances can reveal some crucial insights into the sense of presence, leading to insights on immersion. Finally, after reviewing those works that situate affordances as a determinant of sense of presence, we also acknowledge that this type of study has not succeeded in measuring affordances precisely since the discussions are still derived from the presence measurement. Only 6 out of 17 studies attempt to directly measure affordances and break down the elements of affordances. Therefore, it is worthwhile to note that almost a quarter of the selected studies still utilise presence metrics to study correlates of perceived affordances.

### New wave of affordances-based measurements in VREs

In general, seven studies formulate their own affordance-based measurements(Djebbara et al. 2019; Grechkin et al. 2014; Leyrer et al. 2015; Meagher and Marsh 2015; Readman et al. 2021; Steffen et al. 2019; von Castell et al. 2021) and three studies adopt interdisciplinary theories outside psychology to explore ways to measure affordances (Djebbara et al. 2019; Hameed and Perkis 2021; Memikoğlu and Demirkan 2020). To narrow the scope of the discussion, in this subsection, we will focus on studies that formulate novel metrics to measure correlates of perceived affordances and studies that adopt existing interdisciplinary theories to study affordances.

The first theme discusses studies that conduct VR simulations and formulate novel metrics to study affordances. In the previous subsection, we have learned that one of the most common approaches to measure correlates of perceived affordances is to adopt Warren and Whang’s (1987) measurement of shoulder rotation when participants walk through apertures. Apparently, in the past decade, many researchers have started to formulate a new approach (the list can be seen in Table 7) to study affordances by modifying Warren and Whang’s (1987) aperture simulation (Djebbara et al. 2019; Grechkin et al. 2014) and looking at different cues of perceived affordances. Such cues vary from decision making, task completion, affective responses, bodily movement other than shoulders, as well as brain signals.

**Table 7:**
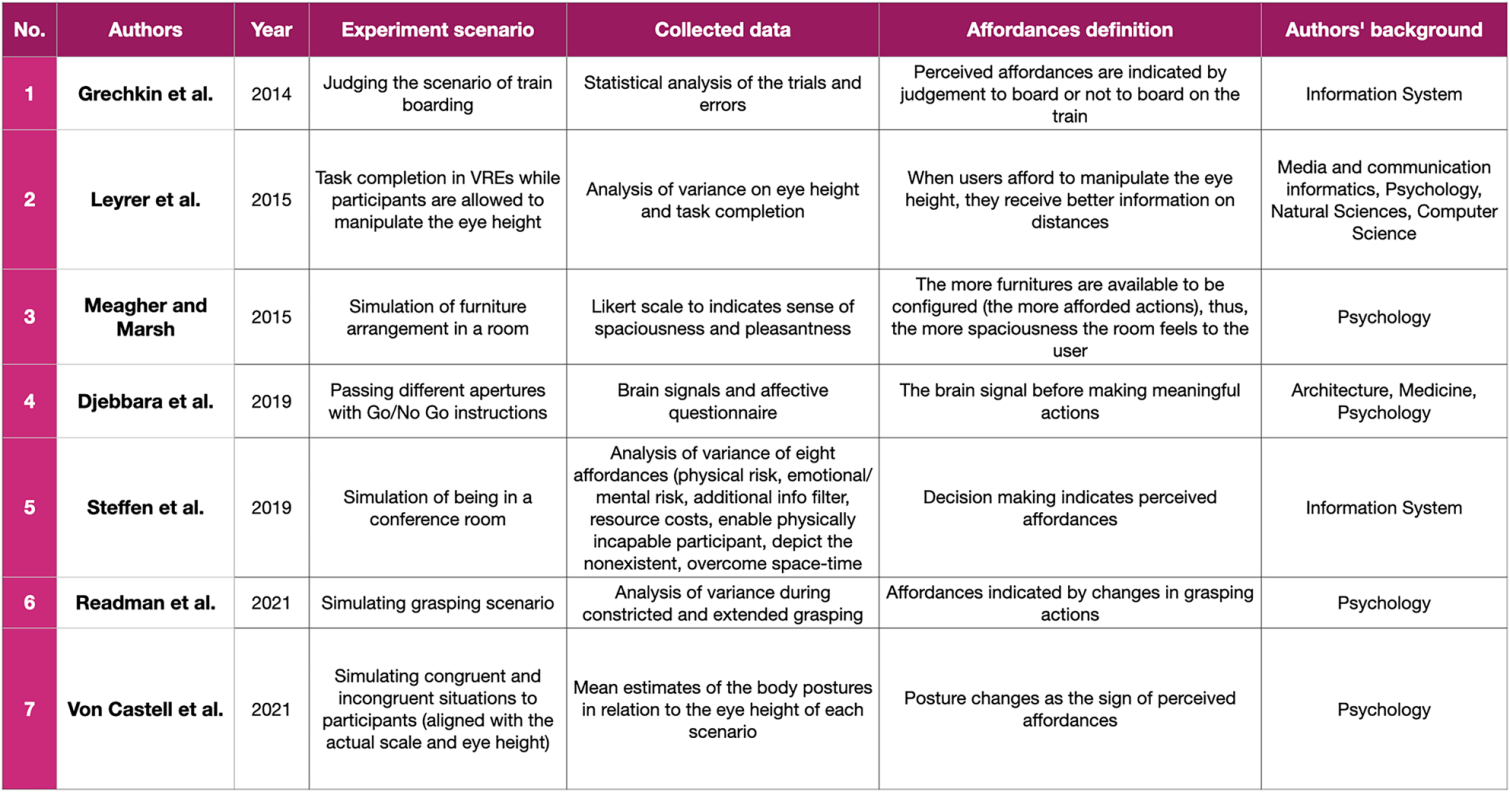
Studies that formulate new approaches to assess correlates of perceived affordances

While such cues aim to explain the direct relationship between humans and environments, their practical interpretation of affordances differs. Thus, it seems to be that there are various dimensions of affordances. For instance, the studies conducted by Grechkin et al. (2014), Leyrer et al. (2015), Readman et al. (2021), and Von Castell et al. (2021) involve a more straightforward scenario where specific actions such as task completion indicate perceived affordances. Then, bodily movement changes are calculated to see how VR users interact with the VREs. Meanwhile, some studies aim to unfold the affective dimensions of affordances. These studies recognise affordances as the ability to sense particular feelings in spaces (Meagher and Marsh 2015) or the ability to anticipate actions (Djebbara et al. 2019). As another example, some studies propose decision-making as an indicator of perceived affordances. Such studies even encompass both physical and affective dimensions of affordances. Although these three types of studies have contributed to enriching the discussion on affordance-based measurements, it seems that there is a prerequisite to continue the discussion on the exact meaning of affordances and related terms.

The second theme covers three studies that adopt interdisciplinary theories to measure affordances. The first study by Djebbara et al. (2019) adopts the concept of “transitions” from architecture to formulate further the way to measure correlates of perceived affordances. According to Djebbara et al. (2019), perceived affordances are denoted by human brain signals before making meaningful actions. Then, the second study by Memikoglu and Demirkan (2020) proposes that understanding the way VR users perform navigation and wayfinding in VREs can help researchers analyse affordances. This study also combines several existing metrics such as Presence Questionnaires (Regenbrecht and Schubert 2021), Sense of Direction Scale (Hegarty et al. 2002), computer aversion index, as well as Computer Experience Questionnaire. Lastly, the third study by Hameed and Perkis (2021) adopts four types of affordances from Hassenzahl’s models (2011) and then creates questionnaires and observations based on the models. These three studies have some interesting similarities, such as (1) the background of the first authors are architecture, and (2) the studies are relatively recent, with dates ranging from 2019 to 2021. Such noteworthy findings could support the critical assumption that it might be beneficial to look at architecture literature to expand the discussion on affordances’ measurements in VREs.

As we discuss the statistical diversity within the selected studies, we have found two significant categories from critically analysing such studies. The first one is the foundational theories of the studies. When observing these studies, we can map the trend of the studies into two streams, namely studies based on Warren and Whang’s seminal work (1987) seminal work (67%) and studies that formulate their theoretical frameworks (33%). The second category is observed by distinguishing the research approach. By analysing the research approach of each study, we have identified two research streams, such as implementing pure quantitative approaches (50%) and adopting mixed-methods approaches (50%). We will look at each study according to each category in Subsection 4. At this point, it is worthwhile to note that an appreciable amount of the studies refer to Warren and Whang’s affordances examination (1987) and shift our discussion to this seminal work.

### Warren and Whang’s (1987) seminal study

As mentioned earlier, most of the selected studies formulate their studies based on Warren and Whang’s seminal work that measures correlates of perceived affordances from aperture passability. In their study, participants were asked to walk through apertures with varying widths (Warren and Whang 1987). Then, affordances were measured from the shoulder rotation, which indicates a new action resulted from perceived affordances (a change from frontal walking to body rotation). This study has its merits, being the first study that showed how to quantify the concept of affordances. Through their studies, Warren and Whang also proposed several concrete terms to describe affordances, such as passability of the apertures and climbability of the stairs. Furthermore, by translating the concept of affordances into an experiment (Warren and Whang 1987), Warren and Whang also presented the way to break down affordances into analysable units: sequences and transitions. The takeaways from their seminal work have become a departure point for scholars pursuing the topic of affordance-based measurement in physical, mediated, and virtual environments. The following discussion in this subsection will look at studies (Coelho et al. 2014; Djebbara et al. 2019; Lepecq et al. 2009; Rybarczyk et al. 2014) that analyse affordances based on the pivotal work discussed earlier (see Table 8).

**Table 8:**
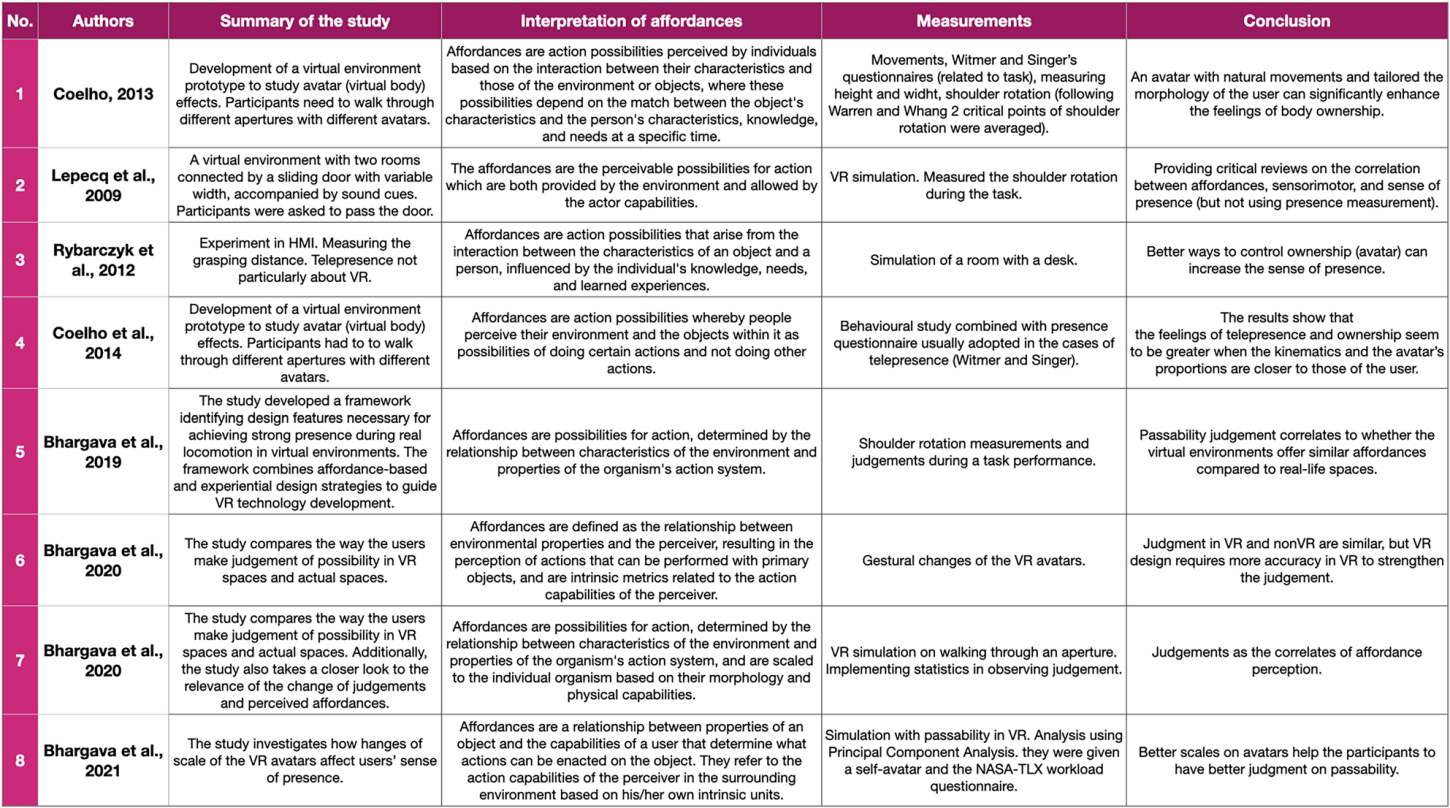
Studies that measure correlates of perceived affordances by adopting Warren and Whang’s concept (1987) of passability

In Table 8, it can be seen that most of the studies explore different hypotheses and objectives. For instance, Lepecq et al. (2009) propose that the shoulder rotation signifies the perceived affordances, indicating a sense of presence. The study was conducted as the virtual version of the Warren and Whang walking through apertures experiment. Therefore, the study implements Warren and Whang’s affordances measurements. Meanwhile, two studies adopt Warren and Whang’s findings on shoulder rotation as affordances cues and modify the environmental settings of the simulation. The first study conducted by Coelho et al. (2014) examines affordances within a long hallway with several apertures. Then, the second study conducted by Rybarczyk et al. (2014) measures affordances within a room with a desk and a chair. Although the virtual scenarios of both studies are modified, both studies reveal significant shoulder rotations when the study participants approach the virtual objects signaling perceived affordances. Finally, the last study of this category (Djebbara et al. 2019) measured brain signals that indicate sensorimotor activities right before participants passed the aperture to investigate the correlation between perceived affordances and meaningful actions. Having reviewed the four studies orientated to the Warren and Whang affordance experiment with apertures, we can distinguish insights that advance the discourse on affordances measurements. At the same time, we need to keep in mind that those studies explore somewhat limited experiment scenarios and very specific types of affordances.

This systematic review has identified two emerging issues within the discourse of affordances measurements: the prevalence of quantitative approaches, particularly post-test quantitative questionnaires, and most of the identified studies develop their experiment designs from Warren and Whang’s aperture experiments. Those issues are problematic for twofold reasons. First, quantitative approaches tend to show generalisable trends or events within a phenomenon but are unable to reveal the phenomenon’s narrative. Second, post-test questionnaires heavily rely on memories, making the data collection not real-time. Third, following one experiment design scenario to discuss affordances is somewhat limiting. Therefore, our next suggestion is to look for alternative techniques to measure affordances from other disciplines investigating the relationship between humans and their built environment. Thus, in the next section, we discuss different techniques to measure affordances in the architecture domain.

## Traditional literature review in architecture

This section critically reviews the three most common architectural techniques for measuring spatial affordances. Such techniques are model-making, movement notation, and creative mapping, selected based on the reviewed literature (Ching 2012; Halprin 1970)the first author’s experiences from studying and teaching in architectural design studio courses. These techniques will be discussed based on literature within the architectural domain.

### Assessing affordances as reading spaces in architecture

In architecture, there is a practice called reading spaces or reading architecture as a way to assess and study the potentials of spaces. This act of reading is not simply a literal reading; instead, reading spaces involves the act of extracting meaning and creating interpretation (Harahap et al. 2018). Typically, the practice of reading spaces takes place prior to the design process, where the architects have the opportunity to visit the building site, observe its potentials, and document their findings through a series of visualisations.

Many prominent architects emphasise the importance of reading spaces as part of the design cycle and share the way they conduct reading space throughout the years. For example, Halprin (1970)^8^ coined the phrase of scoring architecture as an act of understanding the potential of spaces. The act of scoring is beyond understanding the physical quality of space. Perhaps this is why scoring to Halprin involves bodily movement and sensory experience. During his scoring process, Halprin typically spent a fair amount of time on the site just to do nothing and sometimes invited his closest ones to spend time and do activities there. Halprin (1970) further explained that scoring architecture is essentially similar to scoring in music or dance. The scores are documentation of the scoring process, where the scoring is dynamic as a result of human-space engagement. Thus, scoring is a way to assess how spaces evolve, how people move through them, and how experiences unfold dynamically.

**Fig. 2:**
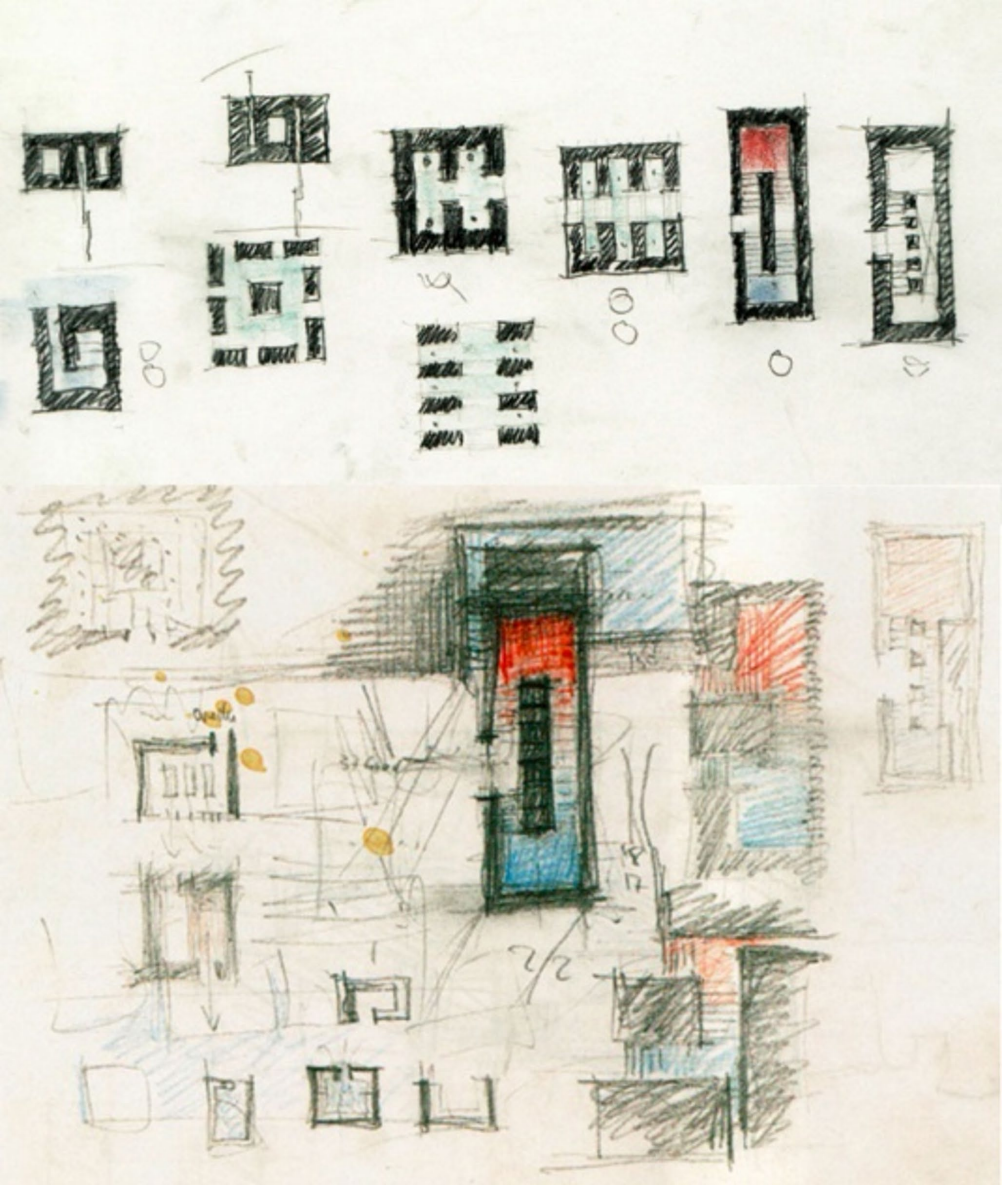
Peter Zumthor’s early sketches of the Therme Vals’ floor plan. n.d., Retrieved from: https://cherylkiwi.wordpress.com/2012/10/23/phenomenology-in-architecture-senses-knowledge-and-awareness/

Another example is Peter Zumthor, who built the Therme Vals in Switzerland. Zumthor (1998) believes that architecture should respond to the landscape. This is reflected from his early sketches of the Therme Vals, where he concepted the spatial division according to the landscape and the atmosphere of the landscape. In his book Thinking Architecture, he elaborates his design philosophy of the Therme Vals where he responded to the invitation from the landscape to create an architecture that was suitable for the landscape spatial atmosphere. Some areas were designed to respond to the warmth that the landscaped had offered, while some areas were designed to respond to the calmness (Fig. 2)^9^. Zumthor (1998) further adds that he favours site-specific solutions over globalised design approaches, so that the design will respect local conditions. Therefore, he suggests that the process of understanding the landscape and its potentials requires patience, deep thought, and engagement between the architects and the landscape (Zumthor 1998).

Both (1970) as well as Zumthor (1998) interchangeably used the term potentials, invitations, offers, or opportunities to describe affordances in space. Therefore, we would like to propose that the practice of reading spaces that has been a part of the architectural design cycle perhaps for decades or even for at least two centuries, is similar to the study of affordances.

### The state of the discussion on affordances in architecture

One of the important literatures on affordances in architecture is Burte’s framework of spatial affordances (Burte 2008). In his work, he used a habitational approach to observe the way people engage with a certain space, which is an art gallery in downtown Mumbai. Based on his approach, there are five dimensions of affordances: occupiability, penetrability, legibility, sociability, and possessability. Occupiability refers to the possibility to dwell in a space. In Burte’s case, that could be the visitors who had paid for the gallery entrance or some local citizens who know the people in the gallery. Penetrability, which is the next phase of habitation, indicates the possibility of entering the space and exceeding the boundaries. This dimension of affordances differs the gallery guests from the locals, as they would not have the same access to enter the facility. The guests could access the special collections and exhibitions, while the locals could not enter. Legibility shares a similar understanding to the Warren and Whang affordances (1987) that refer to physical potentials that humans can recognise. Sociability refers to the possible facilitation of social contacts. Lastly, possessability indicates the possibility that a human can gain a sense of control in space. An example that Burte points out in his writing is that locals who are familiar with the gallery space use the gallery terrace to rest in the afternoon. Burte seems to offer an alternative view to assess a complex concept such as affordances. By taking into account different phases of habitation, Burte explains that our perceptions cannot be separate from social conventions and our embedded knowledge.

Another key research of affordances in architecture is Griffero’s proposal (2019) to consider affordances as atmospheres. He elaborates that the present of affordances are there in the space, whether human is able to perceive them or not. Furthermore, the analogy of atmospheres also brings a new example of affordances as something that we experience immediately when arriving in a space. For example, when being in a holy place, we can feel the sense of serenity leading us to stay in silence. Although Griffero’s proposal does not explain much about how the experience of affordances may differ on individual or social level, his work sheds some light into how to view a complex concept such as affordances.

Having discussed both works from Burte (2008), Griffero (2019), we identified that affordances touch upon different dimensions, such as the physical structure of space that human can recognise, the social dimension, the atmospheres, and the sense of place or the feeling to turn a space to a place. We will use the identified dimensions of affordances to analyse different techniques in architecture to read spaces.

### Technique 1: model-making

Model-making is a common technique in architecture to communicate ideas of spaces through physical forms. This technique is also often associated with the idea of creating a miniature version of a larger piece of architecture. However, understanding models as mere miniatures can sometimes lead to two misconceptions about this technique. First, miniatures are often understood as smaller and simplified versions of the original objects, making them unable to communicate ideas beyond function, such as emotions and feelings. Furthermore, this understanding limits the outcome of the models in which the models have to be visually similar to the original object. Then, the second misconception is that the notion of miniatures often leads to an understanding that designers create miniatures to communicate existing buildings or their final designs. As a matter of fact, model-making is a scoring technique (representing), which means that the technique can also be implemented in communicating abstracted or deconstructed ideas of places or works in progress (Dunn 2010). For instance, model-making can also be utilised to convey the idea of emotional experience as well as the exploratory action possibilities of places instead of simply depicting the tectonic forms of places.

**Fig. 3:**
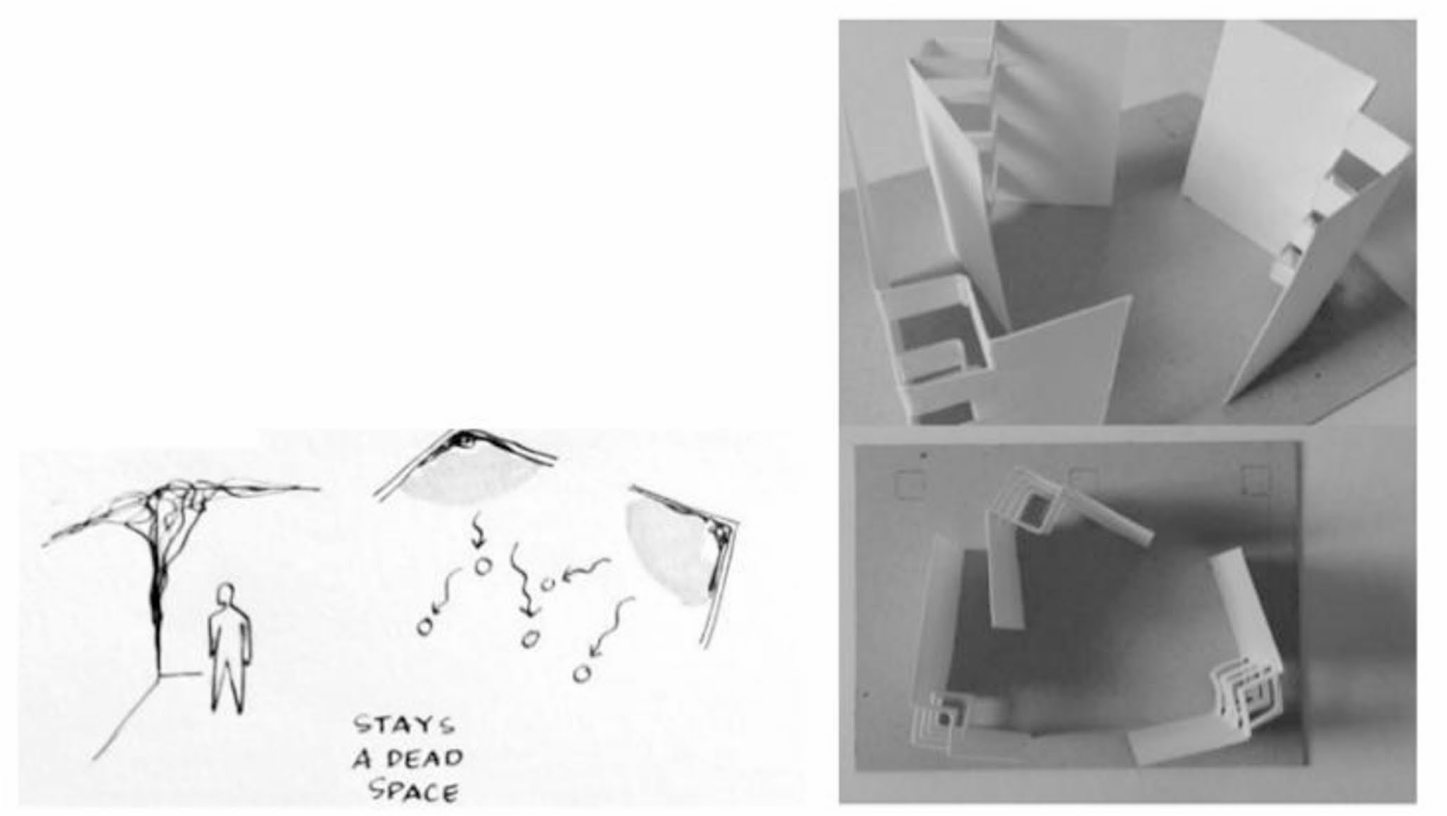
Example of model-making. 2015, Retrieved from https://issuu.com/astaribasuki/docs/model01

Figure 3 below is an example of the atypical use of model-making that indicates insights into spatial affordances. This illustration is taken from a student project who was asked to find an unused space within a building and redesign that space (Basuki 2015). The model in Fig. 3 was created during the initial stage of the design process, in which the student explored the potential of the space by sketching and modeling. When observing the site, the student was interested in the volume of the weed hanging on the corners of the walls. She then explored the volume of the weed and its cavity with her model, which led to her understanding of the shady area in the unused space and how people tend to be under the shades. The shady area became the point of departure of her design. In her design report, she proposed the idea of creating a communal space where people could stand under the shades.

From the example above, we can observe that model-making contains information related to three types of affordances. First, the model-making example reveals the potential actions within the space (standing) and the point at which people tend to do the actions (standing under the shades, so people would tend to walk to the shades). Second, the example above shows the affective dimension of the space, which is the shady area and the preference of people to be under the area. Third, the model also indicates the potential of the space as a communal area, which covers the sense of place and social dimensions of affordances. Thus, from this example of a model-making exercise, we can see the potential of model-making as a technique to study and assess affordances in spaces.

It is fascinating to observe the way in which model-making exercises could reveal many insights related to spatial affordances. Many design theorists have been researching the cognitive dimension of model-making, implying the potential of model-making to reveal information related to spatial affordances. First, model-making enables designers to explore different scales of places. Such scale manipulations grant a sense of authority to the designers, providing them with a better opportunity to observe, understand, maneuver, and manipulate the current model (Busch 1991). Scale explorations in model-making then provide them with more concrete ideas about the action possibilities within the spaces, in other words, the physical-sensorial affordances. Second, model-making allows designers to interact physically with materials while aiming for particular forms. That interaction enables emotions and feelings to materialise into forms, making the model contain affective values. Third, model-making becomes a constant reminder for designers of real-world constraints. By exploring certain forms with physical materials, designers are bound to the basic logic to create forms and structures. In this way, designers will always include the nature of physics in their algorithms, enabling them to constantly include real-world constraints in their design ideas.

After discussing the advantages of model-making as a technique to analyse affordances, it can be seen that this technique can provide detailed insights on affordances in space. However, there are also some limitations to applying model-making in every design case. For example, the technique might not be suitable for measuring spatial affordances in an ample space such as a city. As we all know, in addition to their large scale, cities involve different complex aspects, such as networks of spaces within the city, different buildings with different qualities, and people’s events that influence the temporal organisation of the city. Such complex situations are hard to depict with models; especially design projects tend to follow particular time frames.

### Technique 2: movement-notation (motation)

Movement-notation is one of the architectural techniques to analyse potential actions in a space in a sequential order. In other words, this technique reveal information about which action, happens in which spatial point, and at which order. Since the technique was initially inspired by cinematic and dance scores, movement-notation approaches spaces as a series of events where there are narrative sequences unfolded as we interact with spaces. Some scholars suggest that this approach is similar to storyboarding (Koeck and Roberts 2010). By implementing this technique, spatial designers are forced to see the deconstructed version of spaces, enabling them to identify which particular movement occurs in which particular time and space.

The term movement-notation was originally coined by Halprin (1970). At first, the technique was intended to illustrate possible human movement within designated spaces. Later on, architect Bernard Tschumi (1994) also developed his movement-notation that functions not only as a design exploration tool but also as a transcriber from existing spaces into notation. In principle, both approaches share similarities in the way each approach separates places into three elements, such as time, space, and movement (Corner 2011). However, since we aim to discuss the movement-notation adopted for analysing affordances from existing spaces, we will focus on exploring Tschumi’s examples of movement-notation in this subsection.

In principle, movement-notation consists of two main steps: selecting the base in which the notation will be drawn and drawing the notation to illustrate the interrelation between space, time and movement. Spatial designers mostly use plan, section, or split axonometry drawings. Sometimes, designers even use photographs as the base of the notation. After finding the appropriate drawing as the notation base, spatial designers draw the notation, deconstructing space, time, and movement. In the early application of motation, many architects implemented Laban notation to illustrate the movement in space. Then, they adopted other techniques, such as layering or storyboarding, in order to highlight the element of time. Later on, Tschumi and other architects created their notation according to the narrative of the project. As an example, Tschumi continuously adjusts the notation based on the project requirement level of detail. There are times when Tschumi notates the crowd with more intense colours. At the same time, Tschumi also notates human movement figuratively so that the notation can reflect the meanings and emotions behind the movement. Having discussed the creation process of movement-notation, we can claim that this technique can link physical-sensorial affordances and the spatial properties that enables such affordances.

**Fig. 4:**
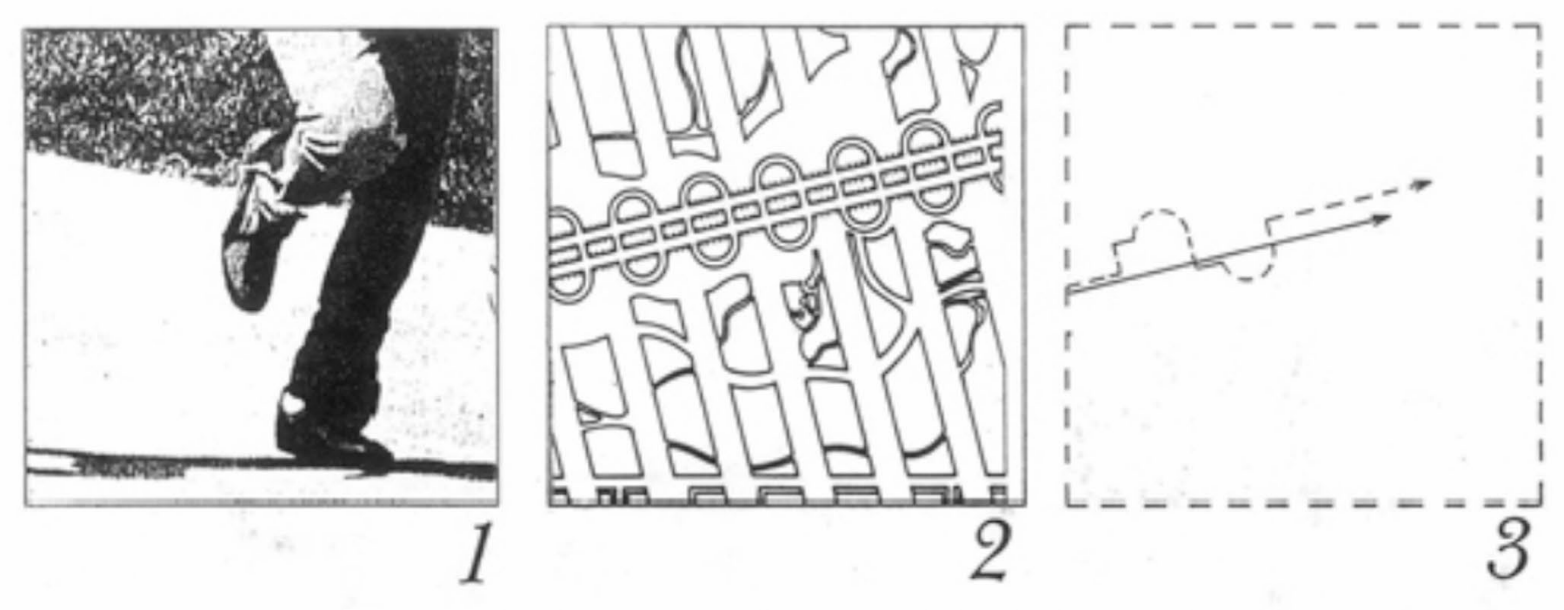
Example of movement-notation. n.d., Retrieved from https://www.tschumi.com/projects/18/. Copyright of Bernard Tschumi Architects

To further discuss the application of movement-notation and possible insights gained from such a technique, we have selected one famous example from the first part of the Manhattan Transcripts (Fig. 4). This movement-notation starts from a picture of the actual space, one of New York’s parks. Instead of exploring a standard spatial scenario, Tschumi explored a scenario of a murder case in which the figure below illustrates the victim running away from danger. After selecting a spatial scenario, Tschumi explored the actual image by implementing several tactics such as tracing, repeating, disconnecting, distorting, fading in, and inserting (Tschumi 1994). Looking at this example, we can observe that Tschumi picked the diagonal line from the photograph, mirrored it, and rebuilt it along different axes. In this case, Tschumi switched from front to top view, providing information about the surrounding components around the crime scene and the route the victim had taken. Although this technique seems to involve the subjective narrative of the person conducting the analysis, this technique enables spatial designers to gain insight into the unconventional functionality of a space. Henceforth, movement-notation has the potential to reveal information related to affordance dimensions, such as the physical structure and the sense of place (Fig. 4).

### Technique 3: creative mapping

Creative mapping has been one of the techniques in architecture to understand the elements that construct our built environment and the way we relate to them. Creative mapping is conducted by communicating the way our body and its multi-modalities experience space through visualisations, texts, numbers, symbols, or notations. Instead of depicting a precise drawing of spaces like conventional mapping, creative mapping focuses on articulating the relationship between space and body, resulting in subjective multimedia maps that inform a place’s material and immaterial qualities (Amoroso 2012). Through creative mapping, architects and other involved participants can better understand spaces as they get to learn the way people inhabit spaces based on subjective bodily experience.

At a glance, creative mapping seems to be similar to movement-notation in the sense that creative mapping requires architectural drawings as the base as well as symbols and notations to pinpoint the insights into spatial affordances. However, creative mapping focuses on revealing the linkage between interesting spots within a space rather than showing potential movement and sequences in space. This focus on highlighting the connection between particular spots encourages architects to identify emerging patterns within the space resulting in insights into atmospheric or place affordances.

As mentioned above, in principle, a creative mapping consists of selecting architectural drawings as its base, marking the essential spots, and then creating a connection between them. In practicing creative mapping, Corner (2011) pinpoints four primary techniques in conducting mapping: drift, layering, game-board, and rhizome. The situationists found the first technique of drift in the 1950s. The core idea of this technique is to focus on subjective human experience by utilising walking as the primary mode to explore and interact with spaces. The second technique is layering, commonly used in the plotting stage when the mapper has already identified several spatial scenarios. After acquiring the spatial scenarios, the mapper layers different scenarios in order to identify the relationship between the scenarios and how it influences one another. Through juxtaposing different spatial scenarios, usually, potential affordances and limitations of spaces can be seen on the maps.

The third technique is game-board, where multiple mappers utilise the mapping field as a contested space. When adopting this technique, different stakeholders with different mapping rules create different spatial scenarios in the mapping field. Lastly, the other technique is rhizome, which adopts the rhizome concept introduced by De Leuze and Guattari, in which rhizome has neither a beginning nor an end. When talking about the rhizome, we talk about a large amount of mapping data in which we can only map the fragment. For instance, narrative or performative mapping of big data in which we can only capture the parts as the data grow simultaneously with time.

In creative architectural mapping, the mappers are usually the designers who make the approach active and critical. Designers actively drift spaces and turn the data from their experience into more abstract maps.In this context, mapping is counted as a process of gathering and analysing data in which the produced maps are the results of selection, omission, isolation, distance, and codification (Corner 2014).

**Fig. 5:**
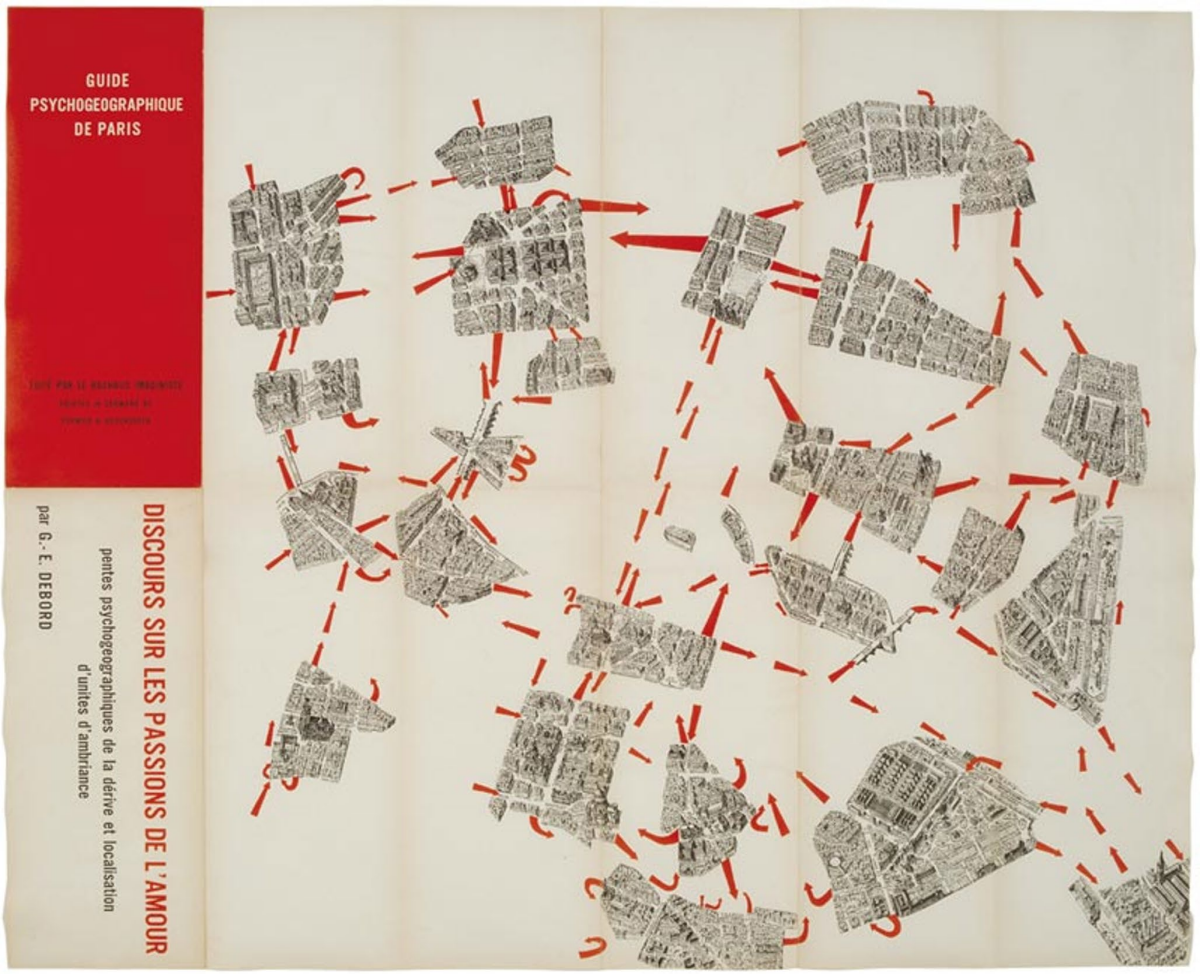
Example of creative mapping. Guide psychogéographique de Paris in 1957. n.d., Retrieved from https://collections.frac-centre.fr/_en/art-and-architecture-collection/debord-guy/guide-psychogeographique-paris-discours-sur-les-passions-l-amour-317.html?authID=53&ensembleID=135. Copyright by François Lauginie

Since the process of creative mapping is seemingly adaptive and flexible, many spatial designers implement this technique to understand the intangible and rare qualities of spaces. One of the excellent examples is the map created by a theorist and situationist named Guy Debord in the 1950s (Corner 2011), which depicted his unique view of experiencing Paris. The philosophy behind this creative map was to provide an alternative experience of the city that opposed the capitalist way to enjoy the city. Thus, instead of experiencing the conventional landmarks, tourist objects, and marketplaces, Debord’s map evoked the way to experience the unexplored urban milieu. He called his maps as a series of psychogeographic guides to provide alternative ways to experience Paris. The maps were derived from his drift experience, where he walked aimlessly around Paris through following his intuition of interesting routes and paths. If we look at the map as illustrated in Fig. 5 below, we can observe that this map contains multiple orders of interesting points that one can follow when exploring Paris.

Through the creative mapping process, it is evident that this technique provides a different way to find captivation within a space and appreciate it. Indeed, this creative map driven by the drifting technique has the potential to explore what a space can offer. By drifting around the city, Debord identified the most scenic route to walk and enjoy the city and the attractive sites based on where locals usually enjoy social interactions. 

Having discussed one of the significant examples of creative mapping, it is noticeable that this technique has its merits in revealing affordance-based information related to human sense of place. By focusing on illustrating connections and routes, creative mapping shows potential events and activities that can occur within spaces. In addition, creative mapping can provide detailed insights into the way people prefer one space over another. Nevertheless, this technique also has limitations because it cannot cover human movements and sequences. Thus, this technique might not be the most appropriate choice to learn about the physical dimension affordances. After reviewing the three most common techniques to analyse spatial affordances in architecture, in the next section we will discuss each technique in the analytical context of this paper.

## Discussion on findings and insights

The purpose of this section is to further discuss techniques to study affordances in VR research and architecture domains, particularly with respect to the merits and drawbacks of the above-mentioned techniques. In this section, we will also discuss the current state of advancement and the potential of each technique to address current research gaps. Since this study involves two types of literature reviews conducted in two different fields, the discussion is organised based on the key takeaways from each review, which are the general statistics of the systematic review in the VR domain and reflection on the takeaways from the architecture techniques.

### General statistics of the systematic review in the VR domain

Through conducting a systematic search and review, we have identified that 36,4% of the studies have come from computer science and information system science. If we look a bit further, 9,1% of the studies also have come from fields that are strongly connected to computer science, which are robotics, electrical engineering, and human-computer interaction. Thus, if we conclude those fields are heavily related, we could say that almost half of the studies have come from domains related to computer science.

**Table 9:**
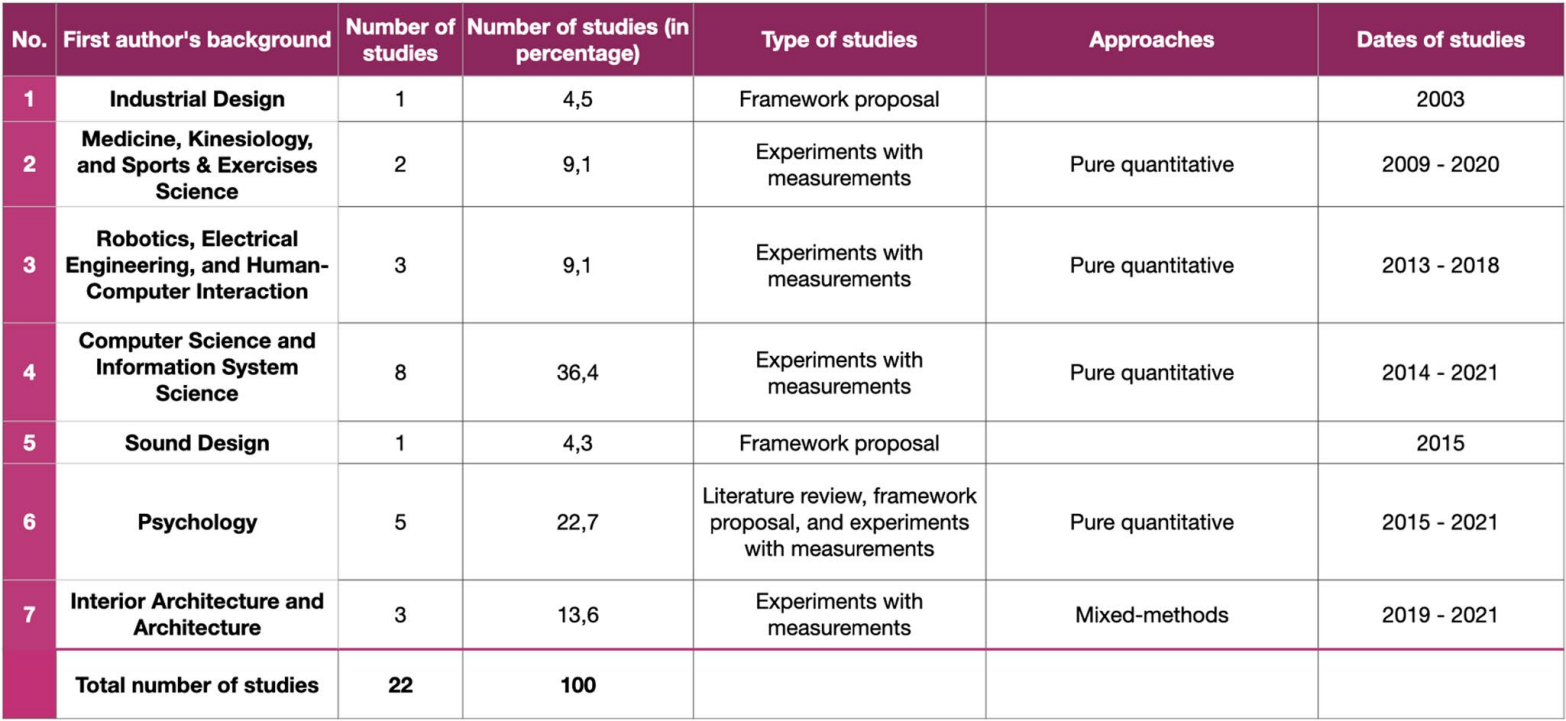
The statistics of the selected studies based on the first Author’s background

When we shift our focus to the dates of the works, we can quickly notice a fascinating emerging insight if we look at the top three domains that have discussed affordances measurements concerning their dates of work. Table 9 indicates that the three most common fields that have contributed to the advancement of the topic are computer science and its related fields, psychology and medicine, kinesiology, and sports and exercise science. Apparently, the field of medicine, kinesiology, sports and exercise science was the field that pioneered the discussion in 2009. Around five years later, computer science and information system science scholars started the conversation, followed by psychology scholars around one year later. Interestingly, beginning in 2019, architecture scholars have joined the discussion on measurement affordances in VR. Around the same time, more experimental studies have emerged to define affordances measurement better. This trend might signify that involving knowledge from architecture could provide additional insights into the advancement of the topic as well as novel approaches to experiment with affordances measurements. Thus, investigating architectural techniques to measure affordances in spaces seems to be the necessary step that contributes to the current discussion.

### Reflection on the traditional review in the architecture domain

Regarding the review of the traditional literature on architecture, this study has evaluated three techniques for studying affordances in spaces, such as modelmaking, motation (movement-notation), and creative mapping. Through the analysis of each technique with regard to the identified dimensions of affordances, we saw that those architecture techniques are able to reveal information on affective dimensions, the narrative of human experience, as well as the connections between meaningful events during human experience in spaces, which cannot be captured through pure quantitative approaches.

However, there is considerable potential emerging from the discussion in which such techniques could be optimised further through actively involving the participants to gain more insights into the created artefacts (models, notations, and maps). Activating participation of participants could mean extending the data collection process by conducting more interviews, presentations, or discussions. Such steps would allow participants to express the narrative of their experience without being stirred with designated questions. In addition, participants could tell their subjective experiences while having the artefacts around them. The artefacts will then help the participants reflect and recall their experience better as they record the critical moments of the participants during the experience in such a personal way.

From the discussion above, we can notice that the techniques to study affordances in architecture tend to emphasise the role of the human as an active agent within the built environment. The above-mentioned techniques enable people to tell their experiences based on their accounts and steer the data collection process. As one additional example, the participants will only be provided with the basic rule of creating models, notations, and maps. Meanwhile, during the data collection process, the participants are free to decide the parts of the experience they would like to express. By recording subjective human experience in a human-centred way, we argue that we could gain more detailed insights into such experience.

## Conclusion

This review brings together different techniques for measuring the affordances of the VR research domain and architecture domain. After completing the systematic review in VR research and the review of the traditional literature in architecture, we have identified four key findings that could be relevant to our research questions.

The first finding is that this review finds that the current advancement of affordance-based measurements is still dominated by scholars in the field of computer science and then by scholars in the field of psychology. Such a finding leans towards our proposal to look for other fields of knowledge and learn about techniques to assess affordances from those other fields.

Our second finding identifies a new wave in research on the affordance-based measurements. In recent years, more mixed-method studies have been conducted to obtain information from behavioral observations in addition to quantitative questionnaires, physical data, and physiological data. Interestingly, this direction is parallel to the presence of more interdisciplinary research that involves architecture. This emerging trend might confirm the relevance of architecture as a field that can contribute to the discussion on the study of affordances, particularly in the context of virtual spaces.

The third finding reveals that techniques to read space in architecture can indicate insights into feelings and emotions in relation to spatial properties such as forms, shapes, or colours. Such a potential can help researchers make sense of the relations between spatial features and perceived affordances.

**Fig. 6:**
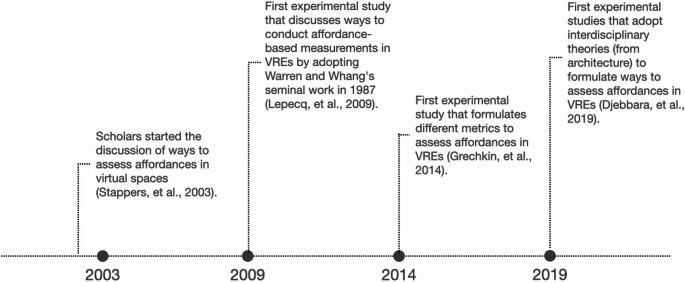
Milestones in the advancement of affordance-based measurements in VR. Figure created by author

The last finding is that this review identifies four events that might have been pivotal in contributing to the topic of affordances measurements. Such events consist of the starting point when VR researchers have started to measure affordances in VR spaces to indicate a successful design (Stappers et al. 2003), the first adaptation of the Warren and Whang (1987) study in a VR space (Lepecq et al. 2009), the first experimental metrics in affordance-based measurements in VR spaces (Grechkin et al. 2014), and the first experimental study that adopts architectural knowledge to measure affordances in a VR space (Djebbara et al. 2019). Such milestones mark the advancement of the topic discussion in VR research (see Fig. 6).

We would like to point out that we have not employed the search process in other literature databases other than Google Scholar. Therefore, there might have been limitations in terms of search returns. At the same time, as the first review that attempts to bring together discussions in the measurements of affordances, this review has raised a discussion that the techniques from architecture might have the potential to complement the existing affordance-based measurements in domains like VR development. Thus, in the future, more literature reviews with more rigorous search, as well as interdisciplinary experiments, will be required to advance the discussion of the topic.

## Footnotes

1 Prior to the origination of the theory of affordances, three several key terms contributed to Gibson’s thinking process (1979). First, Koffka (1935) proposed the term demand character to suggest a property of every object that said what they were. For instance, “…thunder says ‘Fear me’; and a woman says ‘Love me’”. Secondly, the concept of invitation character was proposed by Kurt Lewin (Marrow 1969). The term was originally coined in Germany as Aufforderungscharakter (Brown 1929). Lastly, Lewin further proposed another concept called valence, which suggested “the characters of objects that invited or demanded behaviour” (Marrow 1969, as cited in Gibson 1979).

2 The search was performed on 4 November 2021 at 15.47 EET. The search aimed for relevant studies ranging from 2001 to 2021.

3 In the beginning of the search process, we utilised the strings “measure affordances” AND “virtual reality”. However, we only received 5 returns. Therefore, we attempted the search with slightly modified strings to expect more various returns from any studies that discuss perceived affordances.

4 We did not utilise apostrophe to anticipate any mention of the topic of body in architectural space.

5 Yang et al. (2017) also mentioned in their work that the process model has been adopted from Pickering and Byrne’s (2014) framework.

6 In this article, we use the term of traditional literature review to provide distinction to the systematic review. This traditional literature review refers to a review of literature that is done manually according to a certain set of scope, focus, and theme (Chinn 2021).

7 The domains are referred consecutively from the first authors’ current affiliations, bachelor degrees, PhD degrees, or their research community (audience).

8 While the first author highly values Halprin's critical thinking in articulating different phases of a creative process, the first author does not agree with Halprin's opinion as written in his book page 197 – 201 (1970) regarding the situation in the occupied territory of Palestine back in the 1970s.

9 In hindsight, it is easy to spot the spatial division from the use of contrast colours like red and blue.
